# Tris[(2*E*)-1,3-bis­(4-chloro­phen­yl)triaz-2-en-1-ido-κ^2^ *N* ^1^,*N* ^3^]cobalt(III)

**DOI:** 10.1107/S1600536812020569

**Published:** 2012-05-12

**Authors:** Gang Yu, Shu-Zhong Zhan, Seik Weng Ng

**Affiliations:** a College of Chemistry & Chemical Engineering, South China University of Technology, Guangzhou 510640, People’s Republic of China; b Department of Chemistry, University of Malaya, 50603 Kuala Lumpur, Malaysia; c Chemistry Department, King Abdulaziz University, PO Box 80203 Jeddah, Saudi Arabia

## Abstract

Mol­ecules of the title compound, [Co(C_12_H_8_Cl_2_N_3_)_3_], lie on a threefold rotation axis. The tris-*N*,*N*′-chelated Co^III^ atom, which is located on the threefold rotation axis, shows a distorted octa­hedral coordination.

## Related literature

For tris­(diphenyl­triazenido)cobalt(III), see: Krigbaum & Rubin (1973 ▶).
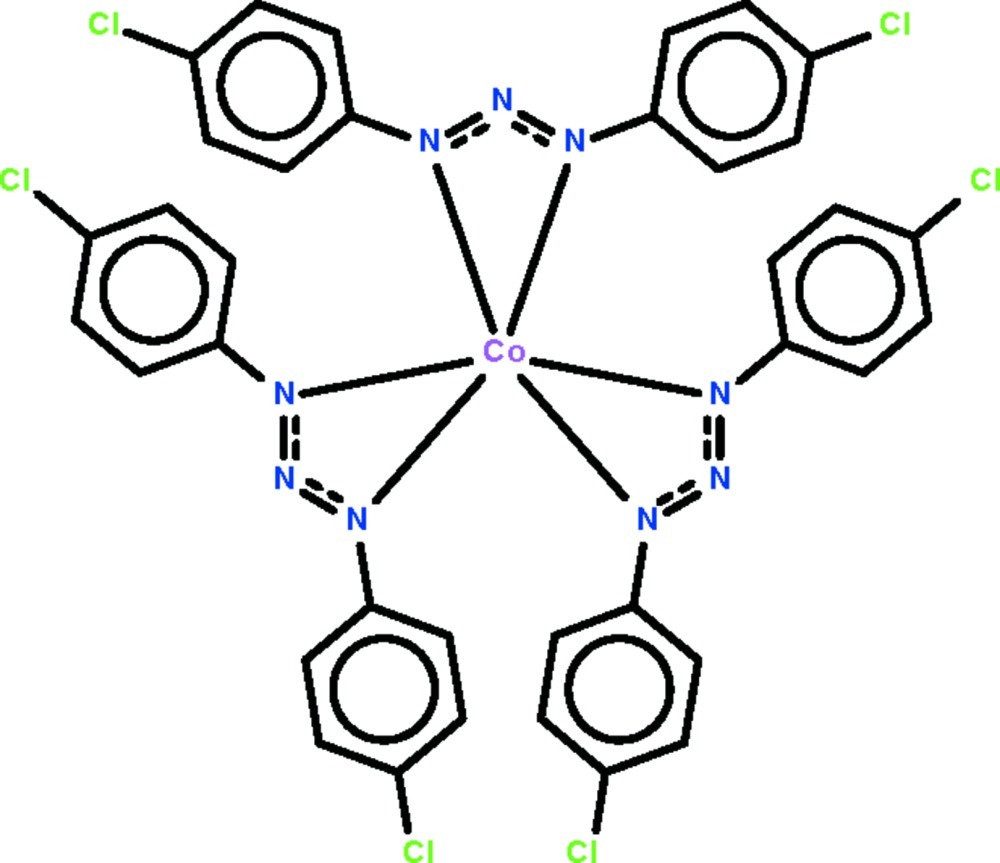


## Experimental

### 

#### Crystal data

[Co(C_12_H_8_Cl_2_N_3_)_3_]
*M* *_r_* = 854.27
Trigonal, 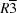

*a* = 19.383 (3) Å
*c* = 17.546 (2) Å
*V* = 5708.5 (10) Å^3^
*Z* = 6
Mo *K*α radiation
μ = 0.91 mm^−1^
*T* = 293 K
0.30 × 0.25 × 0.20 mm

#### Data collection

Bruker SMART APEX diffractometer
Absorption correction: multi-scan (*SADABS*; Sheldrick, 1996 ▶) *T*_min_ = 0.771, *T*_max_ = 0.839
8131 measured reflections
2891 independent reflections
1714 reflections with *I* > 2σ(*I*)
*R*_int_ = 0.053

#### Refinement

*R*[*F* ^2^ > 2σ(*F* ^2^)] = 0.053
*wR*(*F* ^2^) = 0.159
*S* = 1.04
2891 reflections
157 parameters
H-atom parameters constrained
Δρ_max_ = 0.35 e Å^−3^
Δρ_min_ = −0.34 e Å^−3^

### 

Data collection: *APEX2* (Bruker, 2005 ▶); cell refinement: *SAINT* (Bruker, 2005 ▶); data reduction: *SAINT*; program(s) used to solve structure: *SHELXS97* (Sheldrick, 2008 ▶); program(s) used to refine structure: *SHELXL97* (Sheldrick, 2008 ▶); molecular graphics: *X-SEED* (Barbour, 2001 ▶); software used to prepare material for publication: *publCIF* (Westrip, 2010 ▶).

## Supplementary Material

Crystal structure: contains datablock(s) global, I. DOI: 10.1107/S1600536812020569/bt5911sup1.cif

Structure factors: contains datablock(s) I. DOI: 10.1107/S1600536812020569/bt5911Isup2.hkl

Additional supplementary materials:  crystallographic information; 3D view; checkCIF report

## Figures and Tables

**Fig. 1.**
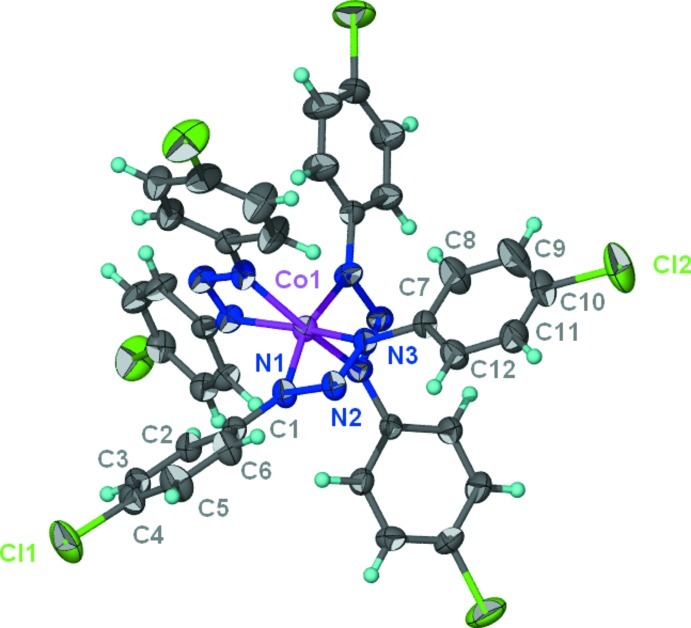
Anisotropic displacement ellipsoid plot (Barbour, 2001) of Co(C12H8Cl2N3)3 at the 50% probability level; hydrogen atoms are drawn as spheres of arbitrary radius. Symmetry-related atoms are no labeled.

**Fig. 2.**
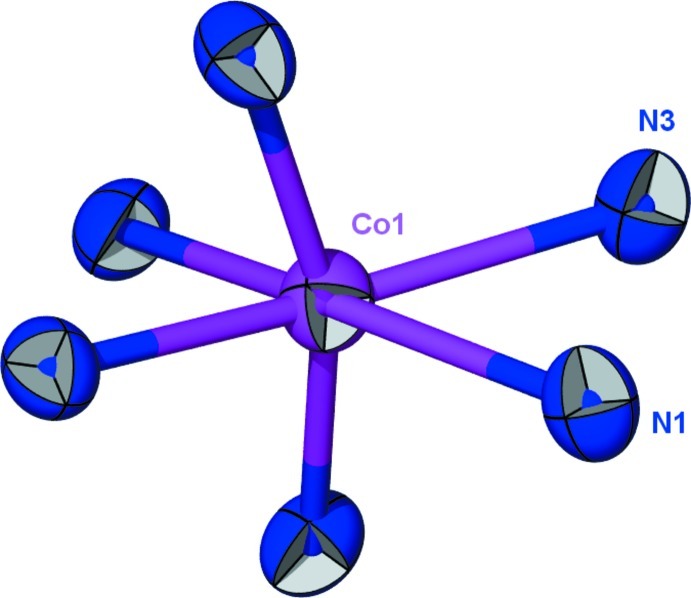
Octahedral coordination geometry of CoIII.

## supplementary crystallographic information

### Comment

The diaryltriazenido anion *N*,*N*'-chelates to a large number of metal atoms; for Co^III^ in particular, three diphenyltriazenido anions chelate to the metal atom to render a trigonal prismatic coordination geometry. Such a geometry appears to be a consequence of the small bite of the chelate (Krigbaum & Rubin, 1973). In chlorine-substituted Co(C_12_H_8_Cl_2_N_3_)_3_ (Scheme I), the metal atom lies on a threefold rotation axis that relates one di(4-chlorophenyl)triazenido chelate to the others (Fig. 1). The Co^III^ atom shows octahedral coordination (Fig. 2). With respect to the delocalized –N–N═N– fragment, one phenylene ring is twisted by 9.5 (3) ånd the other by 21.1 (2) °.

### Experimental

4-Chloroaniline (10 mmol) in water (5 ml) was suspended in 1 *M* hydrochloric acid (30 ml) at 273 K. An aqueous solution (15%) of sodium nitrite (15 mmol) was added dropwise. When the amine had dissolved, a solution of 4-chloroaniline in ethanol (10 mmol) was added at 273 K. The mixture was stirred for 3 h. The reaction mixture was neutralized with a 15% aqueous of sodium acetate to give an orange precipitate. The product was collected and crystallized at 269 K from 9:1 ethyl acetate/n-hexane to give yellow crystals in (65% yield). Calc. for C_12_H_9_Cl_2_N_3_: C 54.16, H 3.41, N 15.79%. Found: C 54.07, H 3.39, N 15.66.%

To a solution of the ligand (0.798 g, 3 mmol) and triethylamine (0.300 g, 3 mmol) in dichloromethane/acetonitrile (30 ml, 1:1) was added cobalt(II) chloride hexahydrate (0.238 g, 1 mmol). The solvent was allowed to evaporate over several days. Red crystals were isolated in 50% yield. Calc. for C_36_H_24_CoN_9_Cl_6_: C 50.59, H 2.81, N 14.76%. Found: C 51.19, H 2.68, N 14.81%.

### Refinement

H-atoms were placed in calculated positions (C—H 0.93 Å) and were included in the refinement in the riding model approximation, with *U*(H) set to 1.2*U*(C).

### Crystal data

**Table:**

[Co(C_12_H_8_Cl_2_N_3_)_3_]	*D*_x_ = 1.491 Mg m^−^^3^
*M**_r_* = 854.27	Mo *K*α radiation, λ = 0.71073 Å
Trigonal, *R*3	Cell parameters from 1211 reflections
Hall symbol: -R 3	θ = 2.6–22.1°
*a* = 19.383 (3) Å	µ = 0.91 mm^−^^1^
*c* = 17.546 (2) Å	*T* = 293 K
*V* = 5708.5 (10) Å^3^	Prism, red
*Z* = 6	0.30 × 0.25 × 0.20 mm
*F*(000) = 2592	

### Data collection

**Table:**

Bruker SMART APEX diffractometer	2891 independent reflections
Radiation source: fine-focus sealed tube	1714 reflections with *I* > 2σ(*I*)
Graphite monochromator	*R*_int_ = 0.053
ω scans	θ_max_ = 27.5°, θ_min_ = 1.7°
Absorption correction: multi-scan (*SADABS*; Sheldrick, 1996)	*h* = −24→20
*T*_min_ = 0.771, *T*_max_ = 0.839	*k* = −24→22
8131 measured reflections	*l* = −22→22

### Refinement

**Table:**

Refinement on *F*^2^	Primary atom site location: structure-invariant direct methods
Least-squares matrix: full	Secondary atom site location: difference Fourier map
*R*[*F*^2^ > 2σ(*F*^2^)] = 0.053	Hydrogen site location: difference Fourier map
*wR*(*F*^2^) = 0.159	H-atom parameters constrained
*S* = 1.04	*w* = 1/[σ^2^(*F*_o_^2^) + (0.0758*P*)^2^ + 0.3669*P*] where *P* = (*F*_o_^2^ + 2*F*_c_^2^)/3
2891 reflections	(Δ/σ)_max_ = 0.001
157 parameters	Δρ_max_ = 0.35 e Å^−^^3^
0 restraints	Δρ_min_ = −0.34 e Å^−^^3^

### Fractional atomic coordinates and isotropic or equivalent isotropic displacement parameters (Å^2^)

**Table:**

	*x*	*y*	*z*	*U*_iso_*/*U*_eq_	
Co1	0.0000	0.0000	0.25642 (4)	0.0361 (3)	
Cl1	−0.04587 (9)	0.28231 (8)	0.51099 (7)	0.0835 (5)	
Cl2	0.34023 (9)	0.12548 (9)	−0.01594 (8)	0.0937 (5)	
C1	0.0269 (2)	0.1483 (2)	0.35169 (17)	0.0349 (8)	
C2	−0.0262 (2)	0.1092 (2)	0.41122 (18)	0.0402 (8)	
H2	−0.0473	0.0547	0.4178	0.048*	
C3	−0.0476 (2)	0.1507 (2)	0.46038 (19)	0.0441 (9)	
H3	−0.0830	0.1245	0.5001	0.053*	
C4	−0.0162 (2)	0.2313 (2)	0.4501 (2)	0.0477 (10)	
C5	0.0370 (3)	0.2714 (2)	0.3925 (2)	0.0581 (11)	
H5	0.0586	0.3261	0.3871	0.070*	
C6	0.0582 (2)	0.2301 (2)	0.3430 (2)	0.0486 (10)	
H6	0.0935	0.2569	0.3034	0.058*	
C7	0.1553 (2)	0.0949 (2)	0.15565 (17)	0.0368 (8)	
C8	0.1422 (3)	0.0343 (3)	0.1071 (2)	0.0630 (12)	
H8	0.0949	−0.0142	0.1102	0.076*	
C9	0.1988 (3)	0.0447 (3)	0.0536 (3)	0.0725 (14)	
H9	0.1893	0.0033	0.0205	0.087*	
C10	0.2671 (3)	0.1136 (3)	0.0490 (2)	0.0547 (11)	
C11	0.2815 (2)	0.1764 (3)	0.0959 (2)	0.0561 (11)	
H11	0.3288	0.2247	0.0914	0.067*	
C12	0.2254 (2)	0.1673 (2)	0.1496 (2)	0.0485 (10)	
H12	0.2347	0.2094	0.1815	0.058*	
N1	0.04549 (18)	0.10269 (17)	0.30269 (15)	0.0398 (7)	
N2	0.10592 (17)	0.13926 (18)	0.25527 (15)	0.0407 (7)	
N3	0.09691 (17)	0.08162 (18)	0.21030 (15)	0.0387 (7)	

### Atomic displacement parameters (Å^2^)

**Table:**

	*U*^11^	*U*^22^	*U*^33^	*U*^12^	*U*^13^	*U*^23^
Co1	0.0336 (3)	0.0336 (3)	0.0410 (4)	0.01679 (17)	0.000	0.000
Cl1	0.0956 (10)	0.0685 (9)	0.0929 (9)	0.0459 (8)	0.0247 (7)	−0.0207 (7)
Cl2	0.0941 (10)	0.0919 (11)	0.0959 (9)	0.0471 (9)	0.0578 (8)	0.0140 (8)
C1	0.037 (2)	0.036 (2)	0.0365 (16)	0.0218 (17)	0.0013 (15)	0.0045 (14)
C2	0.040 (2)	0.034 (2)	0.0469 (19)	0.0193 (17)	0.0047 (16)	0.0030 (15)
C3	0.042 (2)	0.048 (2)	0.0412 (18)	0.021 (2)	0.0072 (16)	−0.0016 (17)
C4	0.051 (2)	0.045 (2)	0.054 (2)	0.029 (2)	0.0028 (19)	−0.0101 (18)
C5	0.075 (3)	0.034 (2)	0.067 (2)	0.028 (2)	0.013 (2)	0.0024 (19)
C6	0.059 (3)	0.036 (2)	0.052 (2)	0.024 (2)	0.0180 (19)	0.0100 (17)
C7	0.036 (2)	0.037 (2)	0.0390 (17)	0.0193 (17)	0.0046 (15)	0.0031 (15)
C8	0.060 (3)	0.041 (2)	0.070 (3)	0.012 (2)	0.025 (2)	−0.004 (2)
C9	0.078 (3)	0.053 (3)	0.074 (3)	0.023 (3)	0.027 (3)	−0.013 (2)
C10	0.057 (3)	0.059 (3)	0.054 (2)	0.033 (2)	0.023 (2)	0.012 (2)
C11	0.040 (2)	0.052 (3)	0.064 (2)	0.014 (2)	0.0089 (19)	0.000 (2)
C12	0.047 (2)	0.041 (2)	0.050 (2)	0.016 (2)	0.0085 (18)	−0.0059 (17)
N1	0.0381 (17)	0.0350 (17)	0.0450 (15)	0.0174 (14)	0.0081 (13)	0.0005 (13)
N2	0.0401 (18)	0.0385 (18)	0.0438 (15)	0.0199 (15)	0.0076 (13)	0.0020 (13)
N3	0.0325 (16)	0.0369 (18)	0.0445 (14)	0.0156 (14)	0.0031 (13)	−0.0061 (13)

### Geometric parameters (Å, º)

**Table:**

Co1—N1	1.909 (3)	C4—C5	1.373 (5)
Co1—N1^i^	1.909 (3)	C5—C6	1.376 (5)
Co1—N1^ii^	1.909 (3)	C5—H5	0.9300
Co1—N3^i^	1.927 (3)	C6—H6	0.9300
Co1—N3	1.927 (3)	C7—C8	1.368 (5)
Co1—N3^ii^	1.927 (3)	C7—C12	1.386 (5)
Co1—N2^ii^	2.441 (3)	C7—N3	1.406 (4)
Co1—N2^i^	2.441 (3)	C8—C9	1.381 (6)
Cl1—C4	1.737 (4)	C8—H8	0.9300
Cl2—C10	1.741 (4)	C9—C10	1.332 (6)
C1—C2	1.394 (4)	C9—H9	0.9300
C1—C6	1.395 (5)	C10—C11	1.377 (6)
C1—N1	1.403 (4)	C11—C12	1.382 (5)
C2—C3	1.378 (5)	C11—H11	0.9300
C2—H2	0.9300	C12—H12	0.9300
C3—C4	1.376 (5)	N1—N2	1.318 (4)
C3—H3	0.9300	N2—N3	1.306 (4)
			
N1—Co1—N1^i^	103.22 (10)	C2—C3—H3	120.3
N1—Co1—N1^ii^	103.22 (10)	C5—C4—C3	121.3 (3)
N1^i^—Co1—N1^ii^	103.22 (10)	C5—C4—Cl1	120.0 (3)
N1—Co1—N3^i^	163.88 (12)	C3—C4—Cl1	118.7 (3)
N1^i^—Co1—N3^i^	64.54 (12)	C4—C5—C6	119.6 (4)
N1^ii^—Co1—N3^i^	90.28 (12)	C4—C5—H5	120.2
N1—Co1—N3	64.54 (12)	C6—C5—H5	120.2
N1^i^—Co1—N3	90.28 (12)	C5—C6—C1	120.3 (3)
N1^ii^—Co1—N3	163.88 (12)	C5—C6—H6	119.8
N3^i^—Co1—N3	103.63 (10)	C1—C6—H6	119.8
N1—Co1—N3^ii^	90.28 (12)	C8—C7—C12	119.3 (3)
N1^i^—Co1—N3^ii^	163.88 (12)	C8—C7—N3	118.7 (3)
N1^ii^—Co1—N3^ii^	64.54 (12)	C12—C7—N3	122.0 (3)
N3^i^—Co1—N3^ii^	103.63 (10)	C7—C8—C9	120.3 (4)
N3—Co1—N3^ii^	103.63 (10)	C7—C8—H8	119.8
N1—Co1—N2^ii^	98.78 (11)	C9—C8—H8	119.8
N1^i^—Co1—N2^ii^	134.58 (11)	C10—C9—C8	120.4 (4)
N1^ii^—Co1—N2^ii^	32.42 (10)	C10—C9—H9	119.8
N3^i^—Co1—N2^ii^	97.33 (11)	C8—C9—H9	119.8
N3—Co1—N2^ii^	135.13 (11)	C9—C10—C11	120.7 (4)
N3^ii^—Co1—N2^ii^	32.14 (10)	C9—C10—Cl2	120.5 (4)
N1—Co1—N2^i^	134.58 (11)	C11—C10—Cl2	118.8 (3)
N1^i^—Co1—N2^i^	32.42 (10)	C10—C11—C12	119.7 (4)
N1^ii^—Co1—N2^i^	98.78 (11)	C10—C11—H11	120.1
N3^i^—Co1—N2^i^	32.14 (10)	C12—C11—H11	120.1
N3—Co1—N2^i^	97.33 (11)	C11—C12—C7	119.5 (4)
N3^ii^—Co1—N2^i^	135.13 (11)	C11—C12—H12	120.3
N2^ii^—Co1—N2^i^	119.993 (4)	C7—C12—H12	120.3
C2—C1—C6	118.9 (3)	N2—N1—C1	119.2 (3)
C2—C1—N1	118.0 (3)	N2—N1—Co1	96.6 (2)
C6—C1—N1	123.1 (3)	C1—N1—Co1	141.9 (2)
C3—C2—C1	120.5 (3)	N3—N2—N1	102.6 (3)
C3—C2—H2	119.7	N2—N3—C7	120.3 (3)
C1—C2—H2	119.7	N2—N3—Co1	96.14 (19)
C4—C3—C2	119.3 (3)	C7—N3—Co1	143.3 (3)
C4—C3—H3	120.3		
			
C6—C1—C2—C3	0.3 (5)	N2^i^—Co1—N1—N2	75.96 (18)
N1—C1—C2—C3	−179.0 (3)	N1^i^—Co1—N1—C1	−113.8 (3)
C1—C2—C3—C4	0.1 (5)	N1^ii^—Co1—N1—C1	−6.5 (4)
C2—C3—C4—C5	−1.0 (6)	N3^i^—Co1—N1—C1	−152.7 (4)
C2—C3—C4—Cl1	178.3 (3)	N3—Co1—N1—C1	162.3 (4)
C3—C4—C5—C6	1.4 (6)	N3^ii^—Co1—N1—C1	57.4 (4)
Cl1—C4—C5—C6	−177.8 (3)	N2^ii^—Co1—N1—C1	26.2 (4)
C4—C5—C6—C1	−1.0 (6)	N2^i^—Co1—N1—C1	−123.4 (3)
C2—C1—C6—C5	0.2 (6)	C1—N1—N2—N3	−168.8 (3)
N1—C1—C6—C5	179.4 (4)	Co1—N1—N2—N3	−2.3 (3)
C12—C7—C8—C9	1.1 (6)	N1—N2—N3—C7	−173.3 (3)
N3—C7—C8—C9	−178.9 (4)	N1—N2—N3—Co1	2.3 (3)
C7—C8—C9—C10	0.5 (8)	C8—C7—N3—N2	−176.9 (3)
C8—C9—C10—C11	−1.9 (8)	C12—C7—N3—N2	3.0 (5)
C8—C9—C10—Cl2	177.4 (4)	C8—C7—N3—Co1	10.5 (6)
C9—C10—C11—C12	1.6 (7)	C12—C7—N3—Co1	−169.6 (3)
Cl2—C10—C11—C12	−177.7 (3)	N1—Co1—N3—N2	−1.69 (19)
C10—C11—C12—C7	0.1 (6)	N1^i^—Co1—N3—N2	−106.2 (2)
C8—C7—C12—C11	−1.4 (6)	N1^ii^—Co1—N3—N2	41.0 (5)
N3—C7—C12—C11	178.6 (3)	N3^i^—Co1—N3—N2	−170.04 (19)
C2—C1—N1—N2	−167.4 (3)	N3^ii^—Co1—N3—N2	82.0 (3)
C6—C1—N1—N2	13.3 (5)	N2^ii^—Co1—N3—N2	74.52 (18)
C2—C1—N1—Co1	34.7 (5)	N2^i^—Co1—N3—N2	−138.0 (2)
C6—C1—N1—Co1	−144.5 (3)	N1—Co1—N3—C7	171.9 (4)
N1^i^—Co1—N1—N2	85.6 (3)	N1^i^—Co1—N3—C7	67.4 (4)
N1^ii^—Co1—N1—N2	−167.2 (2)	N1^ii^—Co1—N3—C7	−145.5 (4)
N3^i^—Co1—N1—N2	46.6 (5)	N3^i^—Co1—N3—C7	3.5 (4)
N3—Co1—N1—N2	1.67 (19)	N3^ii^—Co1—N3—C7	−104.4 (3)
N3^ii^—Co1—N1—N2	−103.3 (2)	N2^ii^—Co1—N3—C7	−111.9 (4)
N2^ii^—Co1—N1—N2	−134.4 (2)	N2^i^—Co1—N3—C7	35.6 (4)

Symmetry codes: (i) −*x*+*y*, −*x*, *z*; (ii) −*y*, *x*−*y*, *z*.
